# Improved Tumor-Targeting with Peptidomimetic Analogs of Minigastrin ^177^Lu-PP-F11N

**DOI:** 10.3390/cancers13112629

**Published:** 2021-05-27

**Authors:** Nathalie M. Grob, Roger Schibli, Martin Béhé, Thomas L. Mindt

**Affiliations:** 1 Department of Chemistry and Applied Biosciences, Institute of Pharmaceutical Sciences, ETH Zurich, 8093 Zurich, Switzerland; grobn@mit.edu (N.M.G.); roger.schibli@psi.ch (R.S.); 2 Center for Radiopharmaceutical Sciences ETH-PSI-USZ, Paul Scherrer Institute, 5232 Villigen, Switzerland; 3 Ludwig Boltzmann Institute Applied Diagnostics, Vienna General Hospital, 1090 Vienna, Austria; 4 Department of Inorganic Chemistry, Faculty of Chemistry, University of Vienna, 1090 Vienna, Austria; 5 Department of Biomedical Imaging and Image Guided Therapy, Division of Nuclear Medicine, Medical University of Vienna, 1090 Vienna, Austria

**Keywords:** cholecystokinin-2 receptor, peptidomimetics, minigastrin, molecular imaging, peptide receptor radionuclide therapy

## Abstract

**Simple Summary:**

Several radiolabeled peptides targeting CCK2R-positive types of cancer (such as medullary thyroid cancer and small cell lung cancer) have been reported in the last 25 years, some of which have entered clinical trials. In an effort to improve its tumor-targeting properties, we applied chemical modifications to the backbone of the peptide ^177^Lu-PP-F11N, an analog of minigastrin in clinical trials. The generated radiolabeled peptidomimetics showed significantly improved characteristics in mice bearing CCK2R-positive tumor xenografts, such as higher tumor uptake, slower tumor washout, and increased tumor-to-kidney ratios. These properties make the novel compounds promising candidates for the imaging and therapy of CCK2R-positive tumors and metastases.

**Abstract:**

The cholecystokinin-2 receptor (CCK2R) is an attractive target in nuclear medicine due to its overexpression by different tumors. Several radiolabeled peptidic ligands targeting the CCK2R have been investigated in the past; however, their low stability against proteases can limit their uptake in tumors and metastases. Substitution of single or multiple amide bonds with metabolically stable 1,4-disubstituted 1,2,3-triazoles as amide bond bioisosteres proved a promising strategy for improving the tumor-targeting properties of a truncated analog of minigastrin. In this study, we applied the previously studied structural modifications to improve the pharmacokinetic and pharmacodynamic properties of PP-F11N, a minigastrin analog currently in clinical trials. Novel minigastrins (NMGs) as analogs of PP-F11N with one or two amide bonds substituted by 1,2,3-triazoles were synthesized, radiolabeled with ^177^Lu^3+^, and subjected to full evaluation in vitro (cell internalization, receptor affinity, stability in blood plasma) and in vivo (stability, biodistribution, SPECT/CT imaging). NMGs with triazoles inserted between the amino acids DGlu^10^-Ala^11^ and/or Tyr^12^-Gly^13^ showed a significantly increased cellular uptake and affinity toward the CCK2R in vitro. Resistance against the metabolic degradation of the NMGs was comparable to those of the clinical candidate PP-F11N. Imaging by SPECT/CT and biodistribution studies demonstrated a higher uptake in CCK2R-positive tumors but also in the CCK2R-positive stomach. The peptidomimetic compounds showed a slow tumor washout and high tumor-to-kidney ratios. The structural modifications led to the identification of analogs with promising properties for progression to clinical applications in the diagnosis and therapy of CCK2R-positive neoplasms.

## 1. Introduction

The cholecystokinin-2 receptor (CCK2R) is a target of high interest in nuclear medicine. Several types of cancer (e.g., medullary thyroid cancer (MTC) and small cell lung cancer (SCLC)) have been reported to express the CCK2R with high incidence and density [1,2]. As these types of cancers can remain asymptomatic over long periods of time, patients are often diagnosed only at late and metastasized stages of the disease [3,4]. Typical treatment options such as chemotherapy, surgical resection, and external beam radiation show only a moderate anti-tumor effect, leading to an overall low survival [5,6]. In light of these facts, reliable methods for early diagnosis and efficient treatment options are urgently needed. Several radiolabeled peptide conjugates targeting the CCK2R have been investigated during the past two decades, [7] some of which have meanwhile entered clinical trials [8,9,10,11]. Although remarkable progress has been achieved, two main challenges in the development of CCK2R-targeting radiopeptides remain: First, the accumulation of radioactivity in the kidneys due to renal elimination and tubular reabsorption mechanisms is a major concern for peptide receptor radionuclide therapy (PRRT) as it can cause nephrotoxicity [12,13]. Second, the rapid proteolytic degradation of the radiotracer after intravenous administration reduces the amount of intact conjugate reaching the tumor, thus impairing the uptake of radiolabeled minigastrins in CCK2R-positive tissues, such as a tumor or the stomach [14,15]. These two challenges must be addressed for the development of new radioligands targeting the CCK2R.

With the intention to improve the resistance of radiolabeled minigastrins against enzymatic degradation, we recently evaluated the substitution of single and multiple amide bonds with 1,4-disubstituted 1,2,3-triazoles in the truncated minigastrin analog [Nle^15^]MG11 (DOTA-DGlu^10^-Ala^11^-Tyr^12^-Gly^13^-Trp^14^-Nle^15^-Asp^16^-Phe^17^-NH_2_) [16,17]. It has previously been shown that triazoles are well suited as metabolically stable amide bond bioisosteres in other peptides targeting G-protein-coupled receptors [18,19,20]. Several single or multiple amide-to-triazole substitutions within the backbone of [Nle^15^]MG11 led to outstanding properties of the resulting triazolominigastrins (TZMGs). The modification of position 4 (Trp^14^-*Ψ*[Tz]-Nle^15^) rendered the peptidomimetic highly resistant against enzymatic degradation in vitro. The insertion of a triazole at position 6 (Tyr^12^-*Ψ*[Tz]-Gly^13^) resulted in a 10-fold increased affinity toward the CCK2R, likely due to an additional cation-π interaction of the peptidomimetic with the receptor. Thus, amide-to-triazole substitutions were able to modify both pharmacokinetic (metabolic stability) and pharmacodynamic (receptor binding) properties. Finally, twofold modification of the peptide at positions 6 and 8 (DGlu^10^-*Ψ*[Tz]-Ala^11^ and Tyr^12^-*Ψ*[Tz]-Gly^13^) combined the positive effects on stability and affinity and resulted in the highest tumor uptake in mice bearing CCK2R-positive tumor xenografts.

In this study, we applied these amide-to-triazole substitutions to PP-F11N (DOTA-(DGlu)_6_-Ala^11^-Tyr^12^-Gly^13^-Trp^14^-Nle^15^-Asp^16^-Phe^17^-NH_2_) [21], a minigastrin derivative with five additional DGlu at the N-terminus in comparison to [Nle^15^]MG11. The hexa-D-glutamate linker improves the metabolic stability of PP-F11N without causing high renal uptake observed for the hexa-L-glutamate analog [22]. Results from phase 0 clinical studies indicate that ^177^Lu-labeled PP-F11N can accumulate in tumors of MTC in potentially therapeutic doses [10]. These promising results led to the progression of the radio-conjugate to phase I clinical trials, which are currently ongoing [8]. 

**Figure 1:**
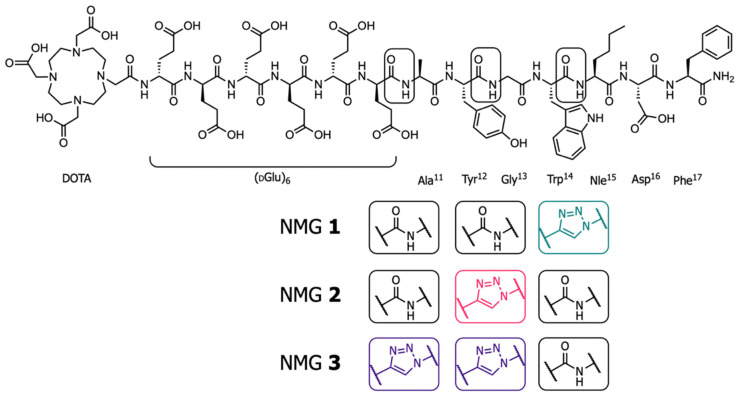
Structure of PP-F11N and NMGs **1**–**3**. The colors of the triazoles correspond to those used in Figure 2, Figure 3 and Figure 4 indicating the position of the amide-to-triazole substitutions (boxes).

Here we report novel minigastrins (NMGs; NMGs **1**–**3**) by placing triazoles in the backbone of PP-F11N at the most promising positions identified by the previous ‘triazole scan’ of [Nle^15^]MG11 (Figure 1) [16,17]. The ^177^Lu-labeled NMGs were subjected to full preclinical evaluation in vitro (IC_50_, cell binding and internalization, plasma stability, logD_pH 7.4_) and in vivo (stability, biodistribution, SPECT/CT imaging), including a side-by-side comparison with PP-F11N.

## 2. Materials and Methods

### 2.1. Synthesis of the NMGs

NMGs **1**–**3** were synthesized and radiolabeled following published procedures (Supporting Information (SI), Scheme S1) [16,17]. Details on the syntheses and analytical data for all compounds can be found in the Supplementary Materials Table S1.

### 2.2. (Radio) Metal Labeling, and Evaluation In Vitro

Procedures for (radio)metal labeling of the DOTA–minigastrin conjugates with ^177^Lu^3+^ and ^nat^Lu^3+^, cell culturing of A431-CCK2R cells [23], cell internalization (including blocking experiments with excess minigastrin (H-LE_5_AYGWMDF-NH_2_)), competition binding assays (IC_50_), stability measurements in human blood plasma, and determinations of logD_pH 7.4_ have been reported previously for the in vitro evaluation of TZMGs and were applied to NMGs without alteration [16,17].

### 2.3. In Vivo Stability

Six-week-old female BALB/c mice were injected with ^177^Lu-labeled NMGs (200 pmol, 100 µL, 2 µM in PBS, 20 MBq, 20 µg/kg) via the tail vein. The animals were sacrificed via CO_2_ asphyxiation, and samples of blood and urine (100 µL) were collected at 10 min post injection (p.i.). For blood samples, proteins were precipitated immediately by addition of CH_3_CN (100 µL), and the samples were centrifuged (3 min, 20238 rcf, rt). The supernatant was diluted with water (1:1), and metabolites were analyzed via γ-HPLC. Urine samples were centrifuged, diluted with water (1:5), and metabolites were analyzed by γ-HPLC. *n* = 2–3 animals per group.

### 2.4. Biodistribution

Procedures for biodistribution studies with ^177^Lu-labeled conjugates (10 pmol, 100 µL, 0.1 µM in PBS, 0.5 MBq, 1 µg/kg) in female CD1-nu mice with xenografts of A431-CCK2R cells at 1, 4, and 24 h p.i., as well as blocking experiments at 4 h p.i. with a 6000-fold excess of minigastrin have been reported previously and were followed without modifications [16]. Statistical analysis was performed via GraphPad Prism using two-sided analysis of variance. *n* = 4 animals per group.

### 2.5. SPECT/CT Imaging

10–14 days prior to the experiment, 6-week-old female CD1-nu mice were inoculated with 5 × 10^6^ A431-CCK2R cells (0.1 mL, 5 × 10^7^ cells/mL in PBS) subcutaneously in both shoulders. The mice were randomly assigned to groups of two animals. On the day of the experiment, ^177^Lu-labeled conjugates (200 pmol, 100 µL, 2 µM in PBS, 20 MBq, 20 µg/kg) were injected via the tail vein. The mice were kept with food and water ad libitum. SPECT scans of 40 min (1 and 4 h p.i., frame duration 60 sec) or 60 min (24 h p.i., frame duration 80 sec) were acquired after an initial CT scan of 7.5 min (CT tube voltage 55 kV, CT tube current 145 µA) with a multi-pinhole NanoSPECT/CTplus (Bioscan Inc., France, and Mediso, Hungary). For the duration of the SPECT/CT scans, mice were kept under anesthesia with a mixture of isoflurane (1.5–2.5%) and oxygen and warmed by a constant air flow of 37 °C. The images were reconstructed with HiSPECT and analyzed in VivoQuant. A post-reconstruction filter was applied (Gauss, full width at half max, 1 mm) to the SPECT images, and the scale was adjusted to visualize important organs and tissues (0.001–0.004 kBq/voxel). *n* = 2 animals per group.

## 3. Results

### 3.1. Synthesis and Radio-labeling

The NMGs were synthesized by solid-phase peptide synthesis in satisfactory yields (11–21%) and high purities (>95%; Supplementary Materials Table S1). Radio-labeling with ^177^Lu^3+^ was achieved in high radiochemical yields and purities of >95% as determined by γ-HPLC and at molar activities of up to 100 MBq/nmol (peptide: nuclide ratio = 7:1; SI, Figures S1–S5).

### 3.2. Evaluation In Vitro

**Figure 2:**
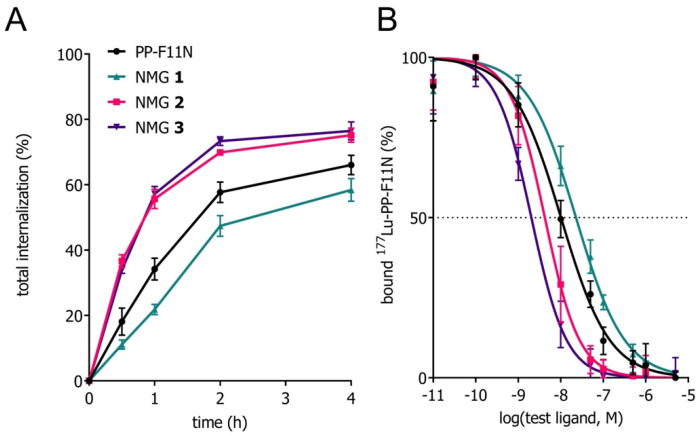
(**A**) Total internalization of ^177^Lu-labeled conjugates (2 nM, 5 kBq) in A431-CCK3R cells over 4 h (*n* = 3–4 in triplicate). (**B**) Receptor displacement of ^177^Lu-labeled CCK2R ligand PP-F11N (2 nM, 5 kBq) by ^175^Lu-labeled NMGs (5E-6 to 1E-11 M) determined on A431-CCK2R cells (*n* = 3 in triplicate). Data points show mean value ± standard deviation.

Table 1 and Figure 2 summarize the physio-chemical properties of the NMGs in vitro in comparison with PP-F11N. NMGs **2** and **3** showed a better affinity toward the CCK2R than the PP-F11N, which correlated with an increased cell internalization at all time points (SI, Figure S6). In contrast, NMG **1** had a higher IC_50_ value compared to PP-F11N, and the cell internalization was reduced. More than 95% of intact radiolabeled conjugates were found for all compounds after 24 h of incubation in human blood plasma in vitro. Internalization was mediated by the CCK2R, as demonstrated by blocking experiments (SI, Tables S2–S5, Figure S7). Minor deviations of logD_pH 7.4_ values were not statistically significant (Supplementary Materials Table S6).

### 3.3. Evaluation In Vivo

**Figure 3:**
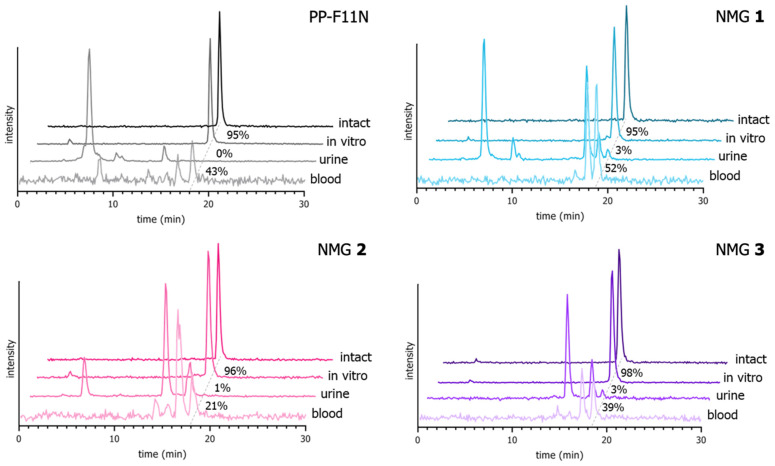
Representative γ-chromatograms of intact ^177^Lu-labeled conjugates and samples of blood and urine at 10 min p.i. in vivo and after 24 h of incubation in plasma in vitro for ^177^Lu-labeled PP-F11N and NMGs show that the compounds are stable in in vitro stability experiments, but are rapidly degraded upon i.v. injection. Furthermore, urine samples show that only negligible amounts of intact radiolabeled peptides are excreted renally. A dashed line shows the retention time of intact conjugates; numbers beside the dashed line stand for mean percentage of intact conjugate (*n* = 2–3).

Metabolites of the ^177^Lu-labeled conjugates were observed in samples of blood and urine of mice at 10 min p.i. of the radiotracers. The amount of intact conjugate (in %) detected in blood decreased in the following order: NMG **1** (52%) > PP-F11N (43%) > NMG **3** (39%) > NMG **2** (21%). Only negligible amounts of intact conjugates were observed in the urine. Representative γ-chromatograms are shown in Figure 3.

**Figure 4:**
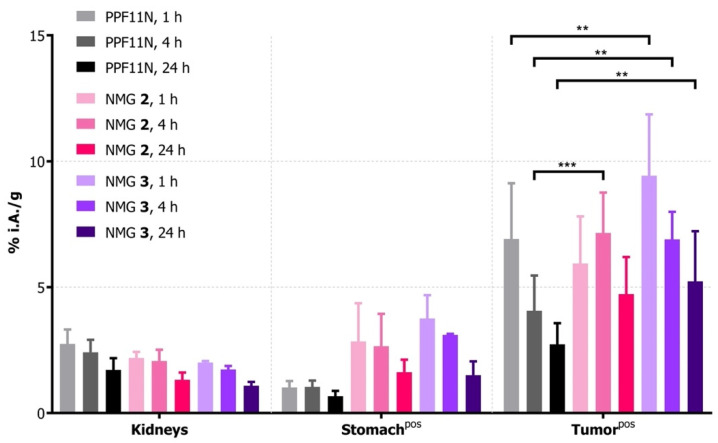
Biodistribution of ^177^Lu-labeled conjugates in relevant organs (kidneys and stomach) and CCK2R-positive tumor xenografts at 1, 4, and 24 h p.i. ** *p* ≤ 0.002, *** *p* < 0.001. Injections: 10 pmol, 100 µL, 0.1 µM, 0.5 MBq, 1 µg/kg. *n* = 4 animals per group (for complete tables of biodistribution and blocking experiments see SI, Tables S7–S12, Figure S8).

Biodistribution experiments were conducted at 1, 4, and 24 h p.i. in mice with CCK2R-positive xenografts. All ^177^Lu-labeled peptide conjugates showed rapid clearance from blood and receptor-negative organs and tissues (for tables of biodistribution and blocking experiments see SI, Figure S8, Tables S7–S12). The uptakes in a CCK2R-positive stomach and tumor were significantly reduced by a co-injection of excess amounts of minigastrin confirming receptor-specific uptake (Supplementary Materials Table S13). Differences in tumor uptakes (% i.A./g) between ^177^Lu-labeled NMGs and PP-F11N were significant at 4 h for NMG **2** (*p* < 0.001) and at all time points for NMG **3** (*p* ≤ 0.002, Figure 4). Uptake in the stomach was higher for NMGs than for PP-F11N, whereas accumulation of radioactivity in the kidneys was slightly—but not with statistical significance—reduced for NMGs in comparison to PP-F11N. The tumor-to-kidney ratios were increased for the triazole-derivatives NMG **2** and **3**, while the tumor-to-stomach ratio was higher for PP-F11N, particularly at 1 h p.i.

**Figure 5:**
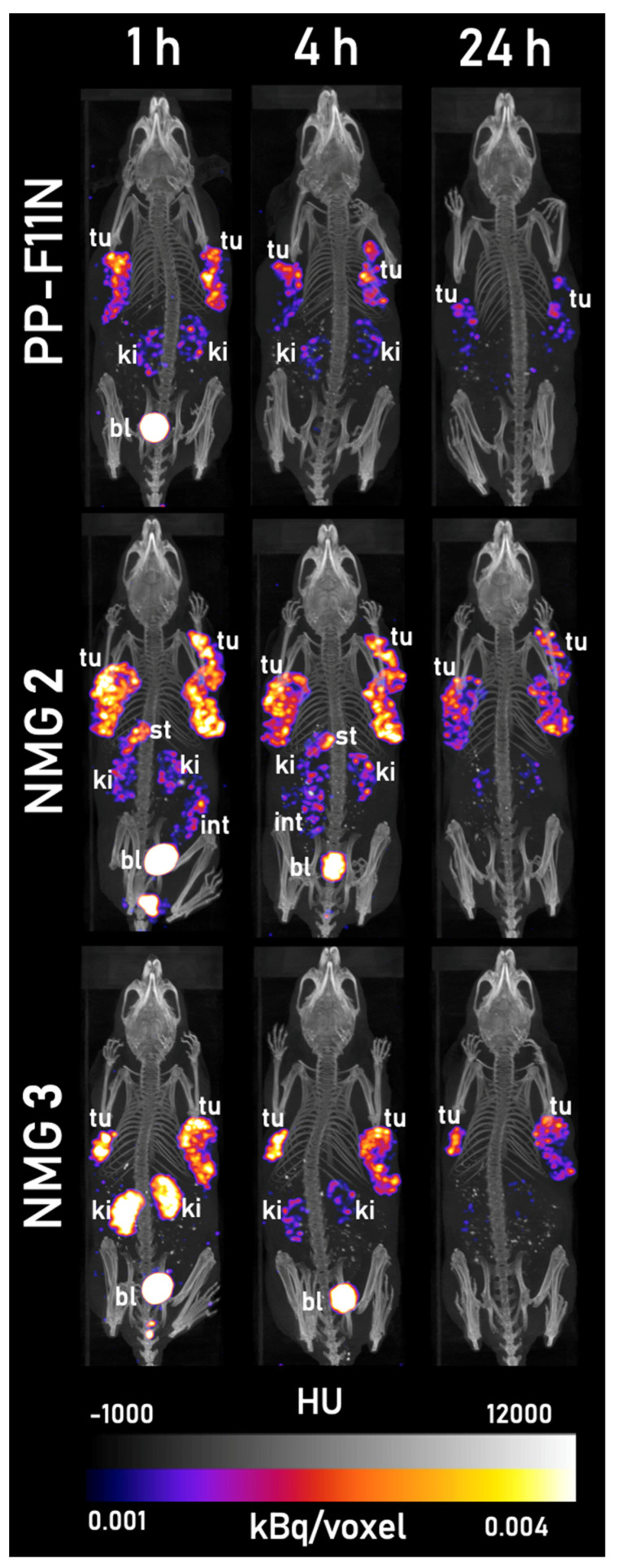
SPECT/CT (MIP) of representative mice with tumor xenografts at 1, 4, and 24 h p.i. of ^177^Lu-labeled PP-F11N (top row), NMG **2** (middle row), and NMG **3** (bottom row). Color gradient represents intensities from 0.001 (dark purple) to 0.004 (light yellow) kBq/voxel of SPECT, black and white gradient refers to −1000 (black) to 12,000 (white) Hounsfield units (HU) of CT. Abbreviations used: tu = tumor xenograft, ki = kidney, bl = urinary bladder, int = intestines. Injections: 200 pmol, 100 µL, 2 µM, 20 MBq, 20 µg/kg. *n* = 2 animals per compound. SPECT/CT of all mice are available in the SI, Figure S9.

SPECT/CT imaging was performed with ^177^Lu-labeled PP-F11N, NMGs **2** and **3** at 1, 4, and 24 h p.i., and it confirmed the results from the biodistribution studies. Representative SPECT/CT images (normalized maximal intensity projection, MIP) are displayed in Figure 5. All radiotracers clearly visualized the tumor xenografts already at 1 h p.i. ^177^Lu-labeled NMGs **2** and **3** showed a higher accumulation of radioactivity in the xenografts at all time points compared to PP-F11N. As a result, the xenografts were still clearly visualized at 24 h p.i. for NMGs **2** and **3**. Due to the renal elimination of the radiotracers, an accumulation of radioactivity was observed in the kidneys and the urinary bladder at 1 and 4 h p.i. and was almost completely eliminated at 24 h p.i. NMG **2** showed low amounts of radioactivity in the gastrointestinal region (CCK2R-positive stomach, intestines) at 1 and 4 h p.i., which was cleared within 24 h.

## 4. Discussion

Targeting of the CCK2R with radiolabeled peptide conjugates is a promising strategy for the diagnosis and endoradiotherapy of tumors overexpressing this receptor. Recent advances in CCK2R-targeting radiopeptides have provided promising results for future use as diagnostic and therapeutic alternatives in the management of patients with MTC and SCLC [8,9,14,25,26]. A general requirement for applications of CCK2R-targeting peptides in nuclear medicine is a high tumor uptake with minimal distribution to healthy organs and tissues. We have recently reported that substitutions of single or multiple amide bonds with metabolically stable 1,4 or 1,5-disubstituted 1,2,3-triazoles improved the tumor-targeting characteristics of the truncated minigastrin analog [Nle^15^]MG11 [16,17,27]. The triazole-containing analogs of [Nle^15^]MG11 exhibited higher affinities toward the CCK2R and increased stability against enzymatic degradation in vitro. In the present work, these structural modifications were applied to PP-F11N, a ^177^Lu-labeled CCK2R-targeting peptide currently under evaluation in phase I clinical trials [8].

Evaluation of the novel NMGs in vitro revealed interesting properties of the radiolabeled peptidomimetics for targeting of the CCK2R (Table 1, Figure 2). NMGs **2** and **3** showed accelerated rates of receptor-specific cell binding and enhanced cell internalization, reaching almost 80% of cell-associated radioactivity after 4 h of incubation (SI, Tables S2–S5). NMG **1** exhibited a reduced cellular uptake after 4 h of incubation as compared to the reference compound PP-F11N. The results were in agreement with the receptor affinities (IC_50_) of the ^nat^Lu-labeled peptidomimetics as determined by competition binding assays. In comparison to PP-F11N (10.1 nM), the IC_50_ values were considerably lower for NMGs **2** and **3** (4.2 nM and 2.0 nM, respectively) and higher for NMG **1** (22.8 nM). In previous studies, we have shown that the substitution of the amide bond at position 6 (Tyr^12^-*Ψ*[Tz]- Gly^13^) of [Nle^15^]MG11 can result in an additional cation-π interaction between the peptidomimetics and the CCK2R, leading to the increased affinity [16]. The observations with NMGs **2** and **3** confirm these results.

Stability studies in vitro using human blood plasma did not show any metabolites for radiolabeled PP-F11N and NMGs after 24 h of incubation (Figure 3), likely due to the stabilizing effect of the N-terminal hexaglutamate moiety in vitro [28]. This is in contrast to previously evaluated triazole-bearing analogs of minigastrin lacking the N-terminal extension [16,17,27]. To evaluate whether the amide-to-triazole substitutions stabilize the analogs of PP-F11N in vivo, we investigated samples of blood and urine of mice at 10 min p.i. of the radiotracers. In contrast to the studies in vitro, metabolites of the ^177^Lu-labeled NMGs were observed in vivo (Figure 3). Membrane-bound proteases (e.g., neutral endopeptidase, angiotensin-converting enzyme) are absent in blood plasma used for in vitro stability studies but are involved in the degradation of various radiolabeled peptides in vivo [15,29,30]. This should be taken into consideration for assessing the metabolic stability of peptidic radiotracers. In samples of blood, 50–80% of the compounds were found metabolized within 10 min; NMG **1** showed the highest stability, PP-F11N and NMG **3** were found to exhibit a similar stability, and NMG **2** had the lowest stability. Negligible quantities of intact radio-conjugates were found in urine samples. These results confirm our previous findings that not all amide-to-triazole substitutions necessarily lead to an increased resistance of the peptidomimetics against metabolic degradation, [16,17] however, can result in the improvement of other biologically relevant characteristics.

^177^ Lu-labeled NMGs **2** and **3**, as well as reference compound PP-F11N, were evaluated in vivo by biodistribution experiments in mice bearing CCK2R-positive tumor xenografts. NMG **1** was not included in the studies due to its inferior receptor affinity and cell internalization in vitro. Previous studies have shown that enhanced stability with reduced receptor affinity leads to minor or no improvement in the biodistribution of the peptidomimetics [16,17]. Therefore, unless the conjugates are functionalized with pharmacokinetic modifiers (preventing them from rapid renal elimination [31]), a favorable receptor interaction outweighs an increase in stability. Already, at 1 h p.i., the ^177^Lu-labeled PP-F11N, and NMG **2** and **3** were cleared from the blood (<0.5% i.A./g), and only low levels of radioactivity were found in CCK2R-negative tissues or organs, resulting in an excellent tumor-to-background ratio. Differences between the NMGs and PP-F11N were observed for the receptor-positive stomach and tumors, reflecting the difference in receptor affinities (IC_50_, Figure 4, Table 1). NMGs **2** and **3** showed an enhanced uptake in the stomach as opposed to PP-F11N. To our delight, the uptake in tumor xenografts was increased accordingly. For example, NMGs **2** and **3** had tumor uptakes that were 1.7 and 1.9 times higher than those of PP-F11N at 24 h p.i. (4.7 ± 1.5, 5.2 ± 2.0 and 2.7 ± 0.8 % i.A./g, respectively). This illustrates the improvement of the CCK2R-targeting properties of NMGs, especially for NMG **3**. Furthermore, the tumor washout for NMGs **2** and **3** was decelerated. For NMG **3**, 56% of the radioactivity accumulated in the tumor at 1 h p.i. was still found in the xenografts after 24 h, whereas only 39% of the radioactivity was retained for PP-F11N (Supplementary Materials Table S11). In addition, the uptake of radioactivity in the kidneys due to the renal excretion of the radiotracers was lower for the NMGs than for PP-F11N. As a result, tumor-to-kidney ratios were higher for NMGs than for PP-F11N at all time points (SI, Tables S6–S11), which is important for applications of the radio-conjugates in PRRT. Another important aspect for therapeutic applications is the uptake of radiolabeled minigastrins in the stomach, which was reported as the dose-limiting organ in clinical trials with PP-F11N [10]. In this regard, the clinical candidate PP-F11N was superior over NMGs **2** and **3**. PP-F11N showed the highest tumor-to-stomach ratios at all measured time points, particularly at 1 h p.i. (Supplementary Materials Table S9–S11). Co-injection of excess minigastrin reduced the uptake in receptor-positive stomach and tumors by more than 50% (SI, Tables S6–S11), hence demonstrating CCK2R-specific uptake.

The potential of ^177^Lu-labeled NMGs **2** and **3** for targeting CCK2R-positive tumors in vivo was further demonstrated by SPECT/CT imaging studies (Figure 5). These studies confirmed the results of the biodistribution experiments. The tumors were clearly visualized by all radiotracers already at 1 h p.i., as were the urinary bladder and the kidneys. The high kidney uptake visible in Figure 5 for NMG **3** at 1 h p.i. was not supported by the results of previous biodistribution studies and may be attributed to a lower water consumption by the animal. The radioactivity in the kidneys and the urinary bladder dropped substantially at 4 h p.i. for all conjugates and was completely cleared at 24 h p.i. NMG **2** also showed low accumulation in the CCK2R-positive stomach and in the intestines at 1 h p.i. only, which was eliminated within 24 h. Both NMGs showed higher uptake of radioactivity in the tumor xenografts at all time points in comparison to the reference compound PP-F11N. The slower tumor washout of NMGs led to higher residual radioactivity in the tumor xenografts at 24 h p.i., providing the possibility for SPECT imaging also at later time points. The good image quality of the SPECT/CT scans with low accumulation in non-targeted tissues could also allow the application of the ^177^Lu-labeled NMGs **2** and **3** for imaging of CCK2R-positive neoplasms via SPECT, although other radionuclides are better suited for imaging applications because of their physical properties (e.g., ^68^Ga, ^111^In). The enhanced uptake and prolonged retention of radioactivity in the tumor, the higher tumor-to-kidney ratios, and the efficient clearance from the kidneys indicate that NMGs **2** and **3** could be suited for both imaging and PRRT. However, the increased uptake of the novel triazole analogs of PP-F11N in the stomach as dose-limiting organ must be considered and requires further investigation.

Several promising CCK2R-targeting radiopeptide conjugates have been reported in the recent years, some of which are currently being investigated in the clinic (PP-F11N, CP04, MGS5) [8,9,25]. In the present work, we evaluated promising NMGs under standardized experimental conditions. To accelerate the clinical translation of minigastrin-based radiopharmaceuticals, a direct, preclinical comparison of the most promising candidates would be desirable. Such comparative studies would streamline and accelerate the efforts of researchers involved in the development of radiolabeled CCK2R-specific probes for routine applications in the diagnosis and endoradiotherapy of CCK2R-positive neoplasms.

## 5. Conclusions

We have successfully applied the amide-to-triazole substitution strategy to the clinically tested minigastrin analog PP-F11N in an effort to improve its performance in molecular imaging and PRRT. The novel peptidomimetic analogs of PP-F11N (NMGs) described in this work exhibit improved affinities toward the CCK2R and increased cell internalization in vitro as well as superior tumor-targeting properties in vivo. The structural modifications of PP-F11N led to an increased uptake of the novel radiotracers in CCK2R-positive xenografts in mice, a slower washout of radioactivity from the tumors, and an improved tumor-to-kidney ratio; however, it also resulted in a reduced tumor-to-stomach ratio that needs to be taken into consideration. In particular, the newly identified lead compound NMG **3** holds promising potential for clinical translation and application in nuclear medicine for the diagnosis and therapy of CCK2R-positive tumors.

## 6. Patents

M.B. and R.S. are the inventors of patent WO201567473. T.L.M., M.B., R.S., and N.M.G. are the inventors of patent WO2019/057445 A1 and patent application EP 20-158 493.5.

## Figures and Tables

**Table 1:** Physio-chemical properties of (radio)metal labeled NMGs in comparison with PP-F11N.

Compound	Sequence	IC_50_ (95% CI, nM) ^a,c^	Internalization 4 h Total (%) ^b,d,f^	Intact Compound In Vitro (%), 24 h, Blood Plasma ^b,d,g^	Intact Compound In Vivo (%), 10 min ^b,d,g^
PP-F11N ^e^	DOTA-(DGlu)_6_-Ala-Tyr-Gly-Trp-Nle-Asp-Phe-NH_2_	10.1	66.0 ± 2.9	98.1 ± 0.9	43.1 ± 2.7
		(8.7–11.8)			
NMG **1**	DOTA-(DGlu)_6_-Ala-Tyr-Gly-Trp-***Ψ*[Tz]-**Nle-Asp-Phe-NH_2_	22.8	58.4 ± 3.5	98.3 ± 0.9	52.0 ± 0.2
		(19.5–26.6)			
NMG **2**	DOTA-(DGlu)_6_-Ala-Tyr-***Ψ*[Tz]-**Gly-Trp-Nle-Asp-Phe-NH_2_	4.2	75.2 ± 2.2	96.3 ± 0.1	21.3 ± 3.0
		(3.6–5.0)			
NMG **3**	DOTA-(DGlu)_6_ -***Ψ*[Tz]-**Ala-Tyr-***Ψ*[Tz]-**Gly-Trp-Nle-Asp-Phe-NH_2_	2.0	76.5 ± 2.8	99.2 ± 0.8	39.3 ± 3.5
		(1.7–2.4)			

^a^ Lu = ^175^Lu. ^b^ Lu = ^177^Lu. ^c^ Data are presented as mean values (95% confidence interval (CI) of nonlinear regression). ^d^ Data are presented as mean values ± standard deviations (SD). ^e^ Data were reported previously [24] and are included for comparison. ^f^ Expressed as % of applied radioactivity. ^g^ In vitro = 24 h incubation time, in vivo = 10 min p.i. of the radiotracer. ***Ψ*****[Tz]** stands for a 1,4-disubstituted 1,2,3-triazole between two amino acids.

## Data Availability

The data presented in this study are available on request from the corresponding author.

## Supplementary Materials

The following are available online at https://www.mdpi.com/article/10.3390/cancers13112629/s1, Synthesis Strategy, General Material, Synthesis of NMGs, Characterization of NMGs, Cell Internalization Data, Biodistribution Data, LogD. Scheme S1: Overview of synthetic strategy, Figure S1: Analytical HPLC chromatogram* of purified NMG **1** (left, t = 11.14 min) and chromatogram from γ-HPLC after radiolabeling with [^177^Lu]Lu^3+^ (right), Figure S2: Representative chromatogram from semi-preparative HPLC of NMG **1** showing separation of diastereomers (NMG **1**(DTrp) at t = 17.47 min, 21.8%: NMG **1**(LTrp) at t = 18.49 min, 78.2%), Figure S3: Analytical HPLC chromatogram of purified NMG **2** (left) and chromatogram from γ-HPLC after radiolabeling with [^177^Lu]Lu^3+^ (right), Figure S4: Representative chromatogram from semi-preparative HPLC of NMG **2** showing separation of diastereomers (NMG **2**(LTyr) at t = 10.31 min, 82.3%: NMG **2**(DTyr) at t = 11.5 min, 17.6%). Figure S5: Analytical HPLC chromatogram of purified NMG **3** (left) and chromatogram from γ-HPLC after radiolabeling with [^177^Lu]Lu^3+^ (right), Figure S6: The IC50 of PP-F11N and NMGs **1**–**3** correlate with the mean total internalization of the compound, Figure S7: Internalization of ^177^Lu-labelled PP-F11N and NMGs over 4 h in presence of a 5000-fold excess of minigastrin for receptor blocking experiments. (n = 3–4 in triplicates), Figure S8: Uptake of all organs for ^177^Lu-labeled PP-F11N and NMGs **2** and **3** at 1, 4, and 24 h p.i., as well as uptake in presence of a 5000-fold excess of minigastrin at 4 h p.i. (n = 4 animals per group), Figure S9: SPECT/CT (MIP) of mice 1-6 with tumor xenografts at 1, 4, and 24 h p.i. of ^177^Lu-labeled PP-F11N (top row), NMG **2** (middle row), and NMG **3** (bottom row). Color gradient represents intensities from 0.001 (dark purple) to 0.004 (light yellow) kBq/voxel of SPECT, black and white gradient refers to −1000 (black) to 12000 (white) Hounsfield units (HU) of CT. Abbreviations used: tu = tumor xenograft, ki = kidney, bl = urinary bladder, int = intestines. Injections: 200 pmol, 100 µL, 2 µM, 20 MBq, 20 µg/kg. n = 2 animals per compound, Table S1: Sequences of the peptide conjugates involved in the study, synthesis yields and data from high-resolution mass spectrometry (HRMS), Table S2: Complete Internalization Data for ^177^Lu-labeled PP-F11N as mean ± standard deviation from n = 3–4 in triplicates, Table S3: Complete Internalization Data for ^177^Lu-labeled NMG **1** as mean ± standard deviation from n = 3–4 in triplicates, Table S4: Complete Internalization Data for ^177^Lu-labeled NMG **2** as mean ± standard deviation from n = 3–4 in triplicates, Table S5: Complete Internalization Data for ^177^Lu-labeled NMG **3** as mean ± standard deviation from n = 3–4 in triplicates, Table S6: summary of logD values of ^177^Lu-labeled NMGs **1**–**3** in comparison to PP-F11N determined at pH 7.4, Table S7: Biodistribution of [^177^Lu]Lu-PP-F11N at 1 h, 4 h, 4 h with blocking, and 24 h as mean ± SD. (n = 4 animals per group), Table S8: Biodistribution of [^177^Lu]Lu-NMG **2** at 1 h, 4 h, 4 h with blocking, and 24 h as mean ± SD. (n = 4 animals per group), Table S9: Biodistribution of [^177^Lu]Lu-NMG **3** at 1 h, 4 h, 4 h with blocking, and 24 h as mean ± SD. (n = 4 animals per group), Table S10: Selected tumor-to-nontumor ratios for ^177^Lu-labeled PPF11N at 1, 4, and 24 h. (n = 4 animals per group), Table S11: Tumor washout of ^177^Lu-labeled PP-F11N, NMGs **2**, and **3**, Table S13: Two-way analysis of variance (ANOVA) of reduction of uptake of radioactivity in receptor-positive tumor and stomach by co-injection of excess amount of minigastrin. Significance and p-values according to Bonferroni multiple comparisons test performed by GraphPad Prism 8.1.2.
