# Supplementary material for: Improved Tumor-Targeting with Peptidomimetic Analogs of Minigastrin 177Lu-PP-F11N
Source: Cancers (Basel). 2021 May 27;13(11):2629. doi: 10.3390/cancers13112629 (PMC8199036; doi:10.3390/cancers13112629)
Supplement: Supplementary file 1 [file cancers-13-02629-s001.zip › cancers-1214692-supplementary.pdf]

Supporting Information:

Improved Tumor-Targeting with Peptidomimetics of Minigastrin Analog ^{177}Lu-PP-F11N

Nathalie M. Grob [1], Roger Schibli [1,2], Martin Béhé [2] and Thomas L. Mindt [3,4,5*]

1 Department of Chemistry and Applied Biosciences, Institute of Pharmaceutical Sciences, ETH Zurich, 8093 Zurich, Switzerland; grobn@mit.edu (N.M.G.); roger.schibli@psi.ch (R.S.)
2 Center for Radiopharmaceutical Sciences ETH-PSI-USZ, Paul Scherrer Institute, 5232 Villigen, Switzerland
3 Ludwig Boltzmann Institute Applied Diagnostics, Vienna General Hospital, 1090 Vienna, Austria
4 Department of Inorganic Chemistry, Faculty of Chemistry, University of Vienna, 1090 Vienna, Austria
5 Department of Biomedical Imaging and Image Guided Therapy, Division of Nuclear Medicine, Medical University of Vienna, 1090 Vienna, Austria
* Correspondence: martin.behe@psi.ch (M.B.); thomas.mindt@univie.ac.at (T.L.M.); Tel.: +41-56-3102917 (M.B.); +43-14040025350 (T.L.M.)
† Current affiliation: Department of Chemistry, Massachusetts Institute of Technology, Cambridge, MA 02139, USA

Table of Contents

SYNTHESIS STRATEGY 2

GENERAL MATERIAL 3

SYNTHESIS OF NMGS 3

CHARACTERIZATION OF NMGS 5

NMG 1 5
NMG 2 5
NMG 3 6

CELL INTERNALIZATION DATA 7

BIODISTRIBUTION DATA 9

LOGD$_{PH\ 7.4}$ 11

REFERENCES 12

Synthesis Strategy

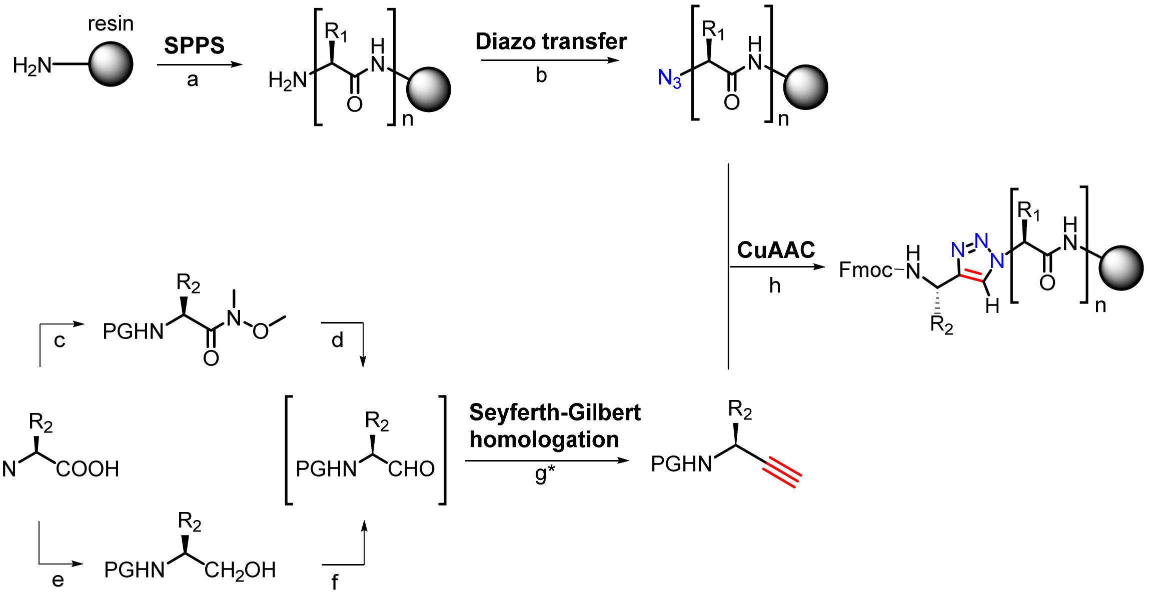

Scheme S1: Overview of synthetic strategy. PG = Boc or Fmoc protecting group; R_1/R_2 = amino acid specific side chain. a) i) amino acids, HATU, DIPEA ii) piperidine; b) imidazol-1-sulfonyl azide HCl, DIPEA; c) BOP, DIPEA, *N,O*-dimethylhydroxylamine; d) DIBAl-H; e) i) NMM, isobutyl chloroformate ii) $NaBH_4$; f) i) DMSO, oxalyl chloride ii) DIPEA; g) MeOH, K_2CO_3, dimethyl-(1-diazo-2-oxopropyl)phosphonate; * if PG=Boc: i)TFA ii) Fmoc-OSu, DIPEA; h) tetrakis(MeCN)Cu(I) PF_6, TBTA, DIPEA. Scheme adapted from Grob *et al.*[1, 2]

Boc	*tert*-butyloxycarbonyl protecting group
Fmoc	fluorenylmethyloxycarbonyl protecting group
HATU	1-[Bis(dimethylamino)methylene]-1H-1,2,3-triazolo[4,5-6]pyridinium 3-oxide hexafluorophosphate
DIPEA	*N,N*-diisopropylethylamine
HCl	hydrochloric acid
BOP	(benzotriazol-1-yloxy)tris(dimethylamino) phosphonium hexafluorophosphate)
DIBAl-H	diisobutylaluminum hydride
NMM	*N*-methylmorpholine
DMSO	dimethyl sulfoxide
TFA	trifluoroacetic acid
OSu	*O*-succinimide
TBTA	tris[(1-benzyl-1H-1,2,3-triazol-4-yl)methyl]amine
SPPS	Solid-phase peptide synthesis
CuAAC	Copper(I)-catalyzed azide–alkyne cycloaddition

General Material

Unless stated otherwise, all chemicals were of analytical grade and used without purification. Instrumentation (HPLC, columns, LC-MS, HRMS, gamma counter) were described in detail in Grob et al., 2020.[1] $^{177}LuCl_3$ was ordered from ITG (itG Lu-177 n.c.a: specific activity (at production) > 3,800 GBq/mg). Human blood plasma (type A^+) was purchased from the cantonal center for blood donation (Aarau, CH). PP-F11N (DOTA-e_6AYGW-Nle-DF-NH_2)[3] and minigastrin (H-LE_5AYGWMDF-NH_2) were synthesized by Peptide Specialty Laboratories GmbH. A431-CCK2R cells were kindly provided by Dr. Luigi Aloj. Female cd1 nu/nu mice and BALB/c mice were purchased from Charles River Laboratories. All animal experiments were performed in compliance with Swiss laws on animal protection and approved by the veterinary office of the canton Aargau under license number AG 75700.

Synthesis of NMGs

In brief, peptides were assembled on solid phase using standard solid-phase peptide synthesis (SPPS) and Fmoc/*t*Bu chemistry following procedures described previously.[1, 2] Once the position for the insertion of the triazole was reached, a diazotransfer reaction using imidiazole-1-sulfoyl azide hydrochloride was carried out with the deprotected N-terminal amine of the peptide to provide the azide. Subsequently, CuAAC of the azide and the corresponding α-amino alkyne (enantiopure after chiral purification or as partially racemized mixture) was accomplished on solid phase using tetrakis(acetonitrile)copper(I) to yield the 1,4-disubstituted 1,2,3-triazole at the desired position(s). The peptide sequences were completed by SPPS and, after the final coupling of the macrocyclic chelator DOTA, conjugates were cleaved from the resin and deprotected. Purification of the crude products by semi-preparative HPLC gave the peptide precursors for subsequent (radio)metal labeling in high purity (>95%). The peptide conjugates were characterized by analytical RP-HPLC and HRMS (SI, Figures S1–S3).

NMG **1** (DOTA-$(DGlu)_6$-Ala-Tyr-Gly-Trp-***Ψ*[Tz]**-Nle-Asp-Phe-NH_2) was prepared by manual SPPS synthesis using commercial DOTA(tris-*t*Bu), Fmoc-DGlu(O*t*Bu)-OH, Fmoc-Ala-OH, Fmoc-Tyr(*t*Bu)-OH, Fmoc-Gly-OH, Fmoc-Nle-OH, Fmoc-Asp(O*t*Bu)-OH, and Fmoc-Phe-OH. Fmoc-Trp(Boc)-CCH was used as a mixture of enantiomers (L:D = 81:19) for the CuAAC, which resulted in 2 diastereomers of the assembled peptide conjugate. The diastereomers were separable by HPLC in a 8:2 ratio using isocratic conditions of 27% MeCN (0.1% TFA) in water (0.1% TFA) over 30 min.

NMG **2** (DOTA-$(DGlu)_6$-Ala-Tyr-***Ψ*[Tz]**-Gly-Trp-Nle-Asp-Phe-NH_2) was prepared by manual SPPS synthesis using commercial DOTA(tris-*t*Bu), Fmoc-DGlu(O*t*Bu)-OH, Fmoc-Ala-OH, Fmoc-Gly-OH, Fmoc-Trp(Boc)-OH, Fmoc-Nle-OH, Fmoc-Asp(O*t*Bu)-OH, and Fmoc-Phe-OH. Fmoc-Tyr(*t*Bu)-CCH was used as a mixture of enantiomers (L:D = 80:20) for the CuAAC, which resulted in 2 diastereomers of the assembled conjugate. The diastereomers were separable by HPLC in a 8:2 using isocratic conditions of 27% MeCN (0.1% TFA) in water (0.1% TFA) over 20 min.

NMG **3** (DOTA-$(DGlu)_5$-DGlu-***Ψ*[Tz]**-Ala-Tyr-***Ψ*[Tz]**-Gly-Trp-Nle-Asp-Phe-NH_2) was prepared by manual SPPS synthesis using commercial DOTA(tris-*t*Bu), Fmoc-DGlu(O*t*Bu)-OH, Fmoc-Ala-OH, Fmoc-Gly-OH, Fmoc-Trp(Boc)-OH,

Fmoc-Nle-OH, Fmoc-Asp(O*t*Bu)-OH, and Fmoc-Phe-OH. Fmoc-Tyr(*t*Bu)-CCH and Fmoc-DGlu(O*t*Bu)-CCH were subjected to chiral HPLC separation prior to the CuAAC. The final peptide conjugate was purified by HPLC using a gradient of 30–40% MeCN (0.1% TFA) in water (0.1% TFA) over 15 min.

Table S1: Sequences of the peptide conjugates involved in the study, synthesis yields and data from high-resolution mass spectrometry (HRMS).

conjugate	sequence	Synthesis yield (%)	HR-MS m/z found (calc.)[a]
NMG **1**	DOTA-$(DGlu)_6$-Ala-Tyr-Gly-Trp-**Ψ[Tz]**-Nle-Asp-Phe-NH_2	20.9	2054.8615 (2054.8614)
NMG **2**	DOTA-$(DGlu)_6$-Ala-Tyr-**Ψ[Tz]**-Gly-Trp-Nle-Asp-Phe-NH_2	19.6	2054.8621 (2054.8614)
NMG **3**	DOTA-$(DGlu)_5$-DGlu-**Ψ[Tz]**-Ala-Tyr-**Ψ[Tz]**-Gly-Trp-Nle-Asp-Phe-NH_2	11.5	2078.8744 (2078.8774)

[a] $[M+H^+]^+$ was observed in all cases

Characterization of NMGs

NMG **1**

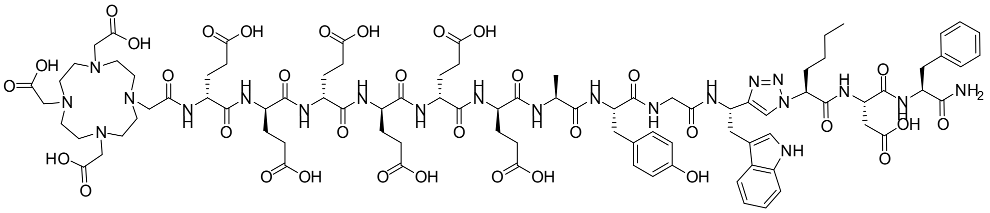

ESI-HRMS calculated for $C_{91}H_{124}N_{21}O_{34}$: 2054.8614; found: 2054.8615

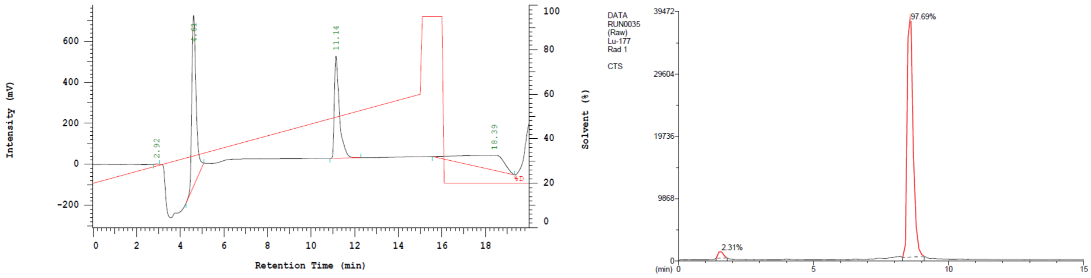

Figure S1: Analytical HPLC chromatogram* of purified NMG **1** (left, t=11.14 min) and chromatogram from γ-HPLC after radiolabeling with ^{177}Lu]Lu^{3+} (right).

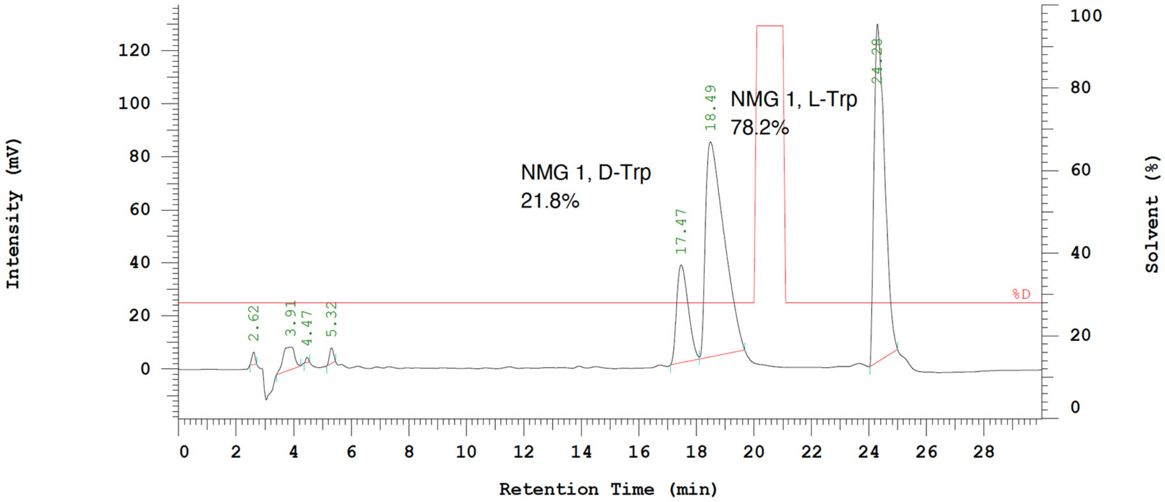

Figure S2: Representative chromatogram from semi-preparative HPLC of NMG **1** showing separation of diastereomers (NMG **1**(DTrp) at t = 17.47 min, 21.8%: NMG **1**(LTrp) at t = 18.49 min, 78.2%).

NMG **2**

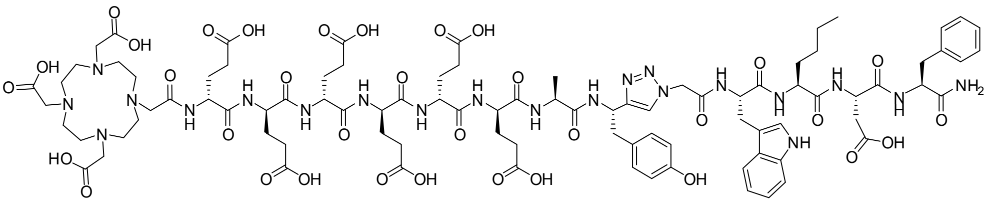

ESI-HRMS calculated for $C_{91}H_{124}N_{21}O_{34}$: 2054.8614; found: 2054.8621

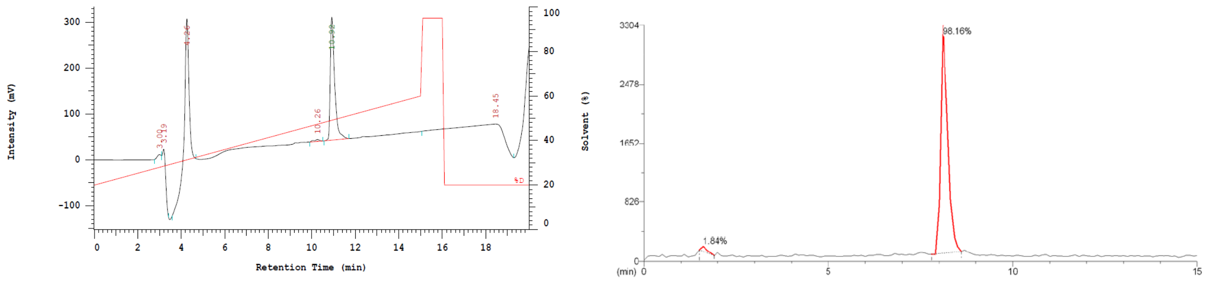

Figure S3: Analytical HPLC chromatogram* of purified NMG **2** (left) and chromatogram from γ-HPLC after radiolabeling with [^{177}Lu]Lu^{3+} (right).

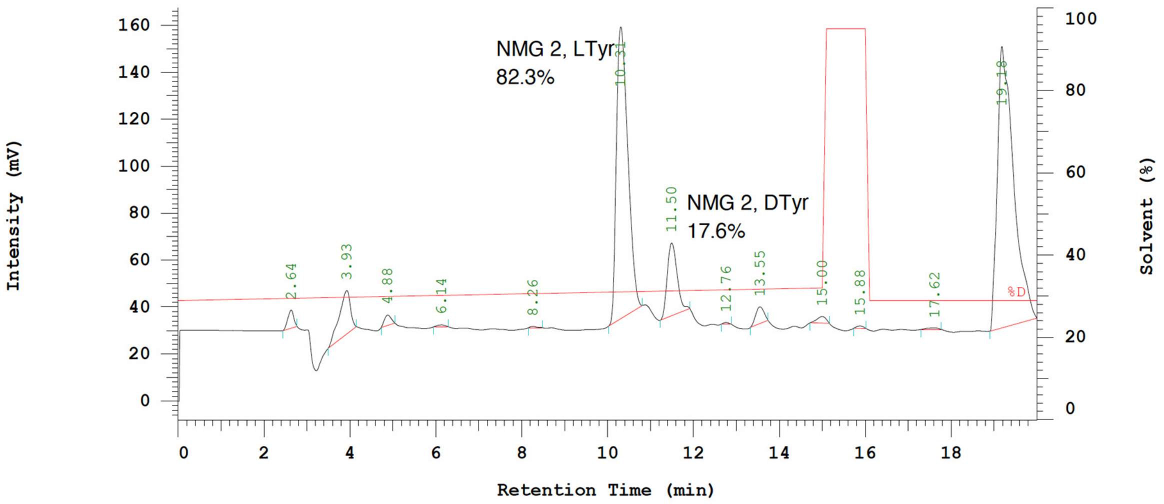

Figure S4: Representative chromatogram from semi-preparative HPLC of NMG 2 showing separation of diastereomers (NMG 2(LTyr) at t = 10.31 min, 82.3%: NMG 2(DTyr) at t = 11.5 min, 17.6%).

NMG **3**

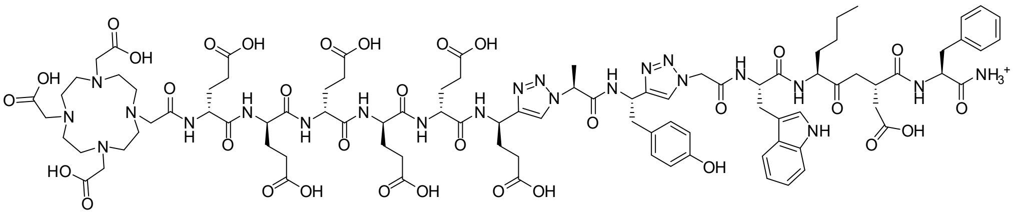

ESI-HRMS calculated for $C_{93}H_{125}N_{22}O_{33}$: 2078.8807; found: 2078.8744

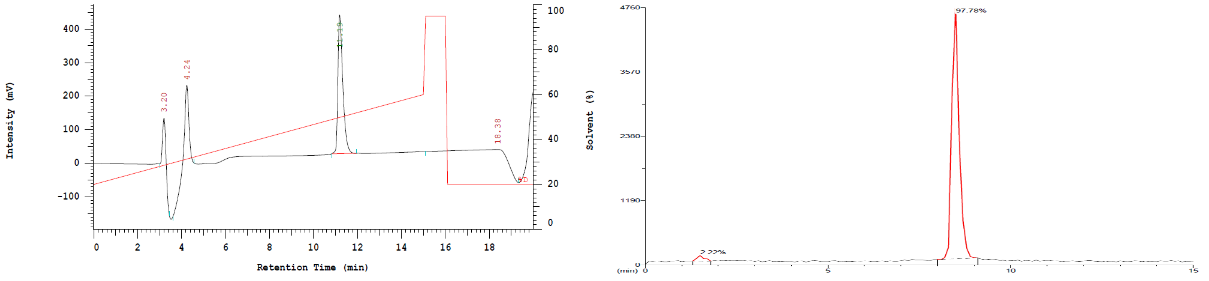

Figure S5: Analytical HPLC chromatogram* of purified NMG **3** (left) and chromatogram from γ-HPLC after radiolabeling with [^{177}Lu]Lu^{3+} (right).

* UV-peaks at t = 4.0–4.5 min results from different composition of mobile phase and sample solvent (injection peak).

Cell Internalization Data

Cell internalization was determined and analyzed as described in Grob, *et al.*, J Med Chem, 2020.[1] Specific values result from values of total internalization or cell binding minus values obtained in presence of a 6000-fold excess of minigastrin.

Table S2: Complete Internalization Data for ^{177}Lu-labeled PP-F11N as mean ± standard deviation from n = 3–4 in triplicates.

Incubation time	membrane-bound			internalized		
	total	blocked	specific	total	blocked	specific
0.5 h	5.4 ± 1.5	0.4 ± 0.2	5.0 ± 1.3	18.1 ± 4.1	0.1 ± 0.1	18.0 ± 4.1
1 h	5.0 ± 1.1	0.3 ± 0.2	4.6 ± 1.0	34.2 ± 3.3	0.2 ± 0.1	34.0 ± 3.3
2 h	3.9 ± 0.5	0.3 ± 0.2	3.5 ± 0.5	57.7 ± 3.1	0.7 ± 0.7	57. 0± 3.3
4 h	3.0 ± 0.4	0.5 ± 0.1	2.5 ± 0.4	66.0 ± 2.9	3.2 ± 1.8	62.9 ± 2.9

Table S3: Complete Internalization Data for ^{177}Lu-labeled NMG **1** as mean ± standard deviation from n = 3–4 in triplicates.

Incubation time	membrane-bound			internalized		
	total	blocked	specific	total	blocked	specific
0.5 h	5.1 ± 0.3	0.3 ± 0.1	4.8 ± 0.3	11.1 ± 4.1	0.02 ± 0.02	11.1 ± 1.4
1 h	4.4 ± 0.1	0.2 ± 0.05	4.2 ± 0.1	21.8 ± 1.6	0.1 ± 0.03	21.8 ± 1.6
2 h	3.9 ± 0.2	0.3 ± 0.06	3.6 ± 0.1	47.4 ± 3.2	0.3 ± 0.2	47.1 ± 3.1
4 h	3.0 ± 0.2	0.4 ± 0.04	2.5 ± 0.1	58.4 ± 3.5	1.6 ± 1.2	56.8 ± 2.5

Table S4: Complete Internalization Data for ^{177}Lu-labeled NMG **2** as mean ± standard deviation from n = 3–4 in triplicates.

Incubation time	membrane-bound			internalized		
	total	blocked	specific	total	blocked	specific
0.5 h	8.4 ± 1.1	0.7 ± 0.1	7.7 ± 1.1	36.7 ± 1.9	0.2 ± 0.03	36.5 ± 1.9
1 h	6.9 ± 0.6	0.7 ± 0.2	6.2 ± 0.8	55.7 ± 2.9	0.4 ± 0.2	55.3 ± 3.0
2 h	4.3 ± 0.3	1.0 ± 0.7	3.3 ± 0.9	69.9 ± 0.9	3.0 ± 3.4	66.9 ± 2.6
4 h	3.4 ± 0.2	1.7 ± 0.7	1.7 ± 1.0	75.2 ± 2.2	13.1± 10.3	62.0 ± 8.1

Table S5: Complete Internalization Data for ^{177}Lu-labeled NMG **3** as mean ± standard deviation from n = 3–4 in triplicates.

Incubation time	membrane-bound			internalized		
	total	blocked	specific	total	blocked	specific
0.5 h	7.7 ± 0.8	0.9 ± 0.3	6.8 ± 1.0	33.9 ± 1.1	0.4 ± 0.3	33.5 ± 1.1
1 h	6.4 ± 0.3	0.9 ± 0.3	5.5 ± 0.4	57.2 ± 2.3	0.7 ± 0.3	56.5 ± 2.1
2 h	4.0 ± 0.6	1.8 ± 1.3	2.2 ± 1.3	73.4 ± 1.3	5.5 ± 3.9	67.9 ± 3.4
4 h	3.2 ± 0.3	2.4 ± 0.6	0.8 ± 0.6	76.5 ± 2.8	18.4 ± 4.3	57.7 ± 4.2

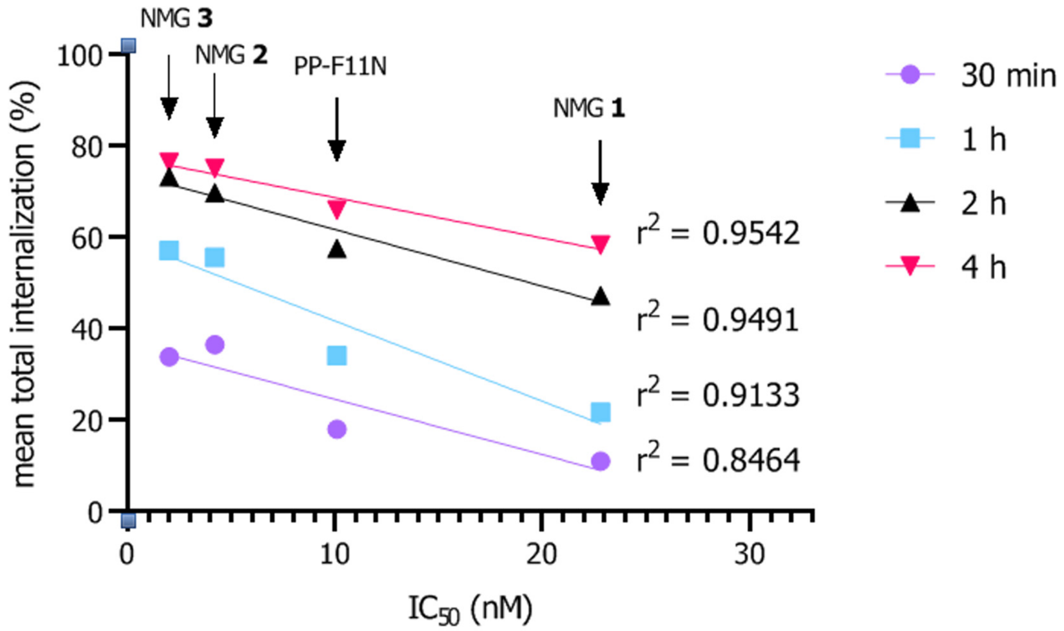

Figure S6: The IC_{50} of PP-F11N and NMGs **1**–**3** correlate with the mean total internalization of the compounds.

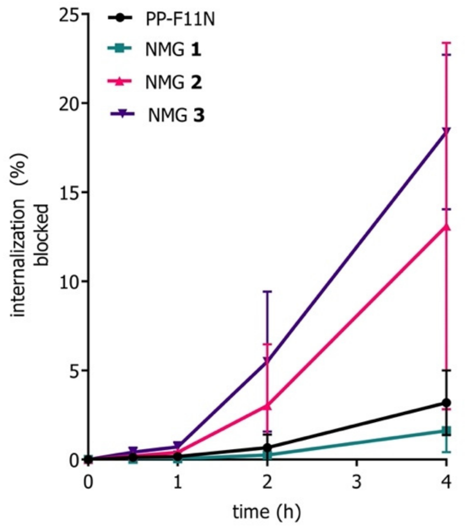

Figure S7: Internalization of ^{177}Lu-labelled PP-F11N and NMGs over 4 h in presence of a 5000-fold excess of minigastrin for receptor blocking experiments. (n = 3–4 in triplicates)

Blocking experiment: In presence of a 6000-fold excess of minigastrin (H-Leu-Glu-Glu-Glu-Glu-Glu-Tyr-Gly-Trp-Met-Asp-Phe-NH_2), the internalization of ^{177}Lu-labeled PP-F11N and NMGs was successfully reduced to ≤5% for all conjugates up to 2 h after the start of the experiment, showing that the cell internalization is specifically mediated by the CCK2R. After 4 h, blocking was unsuccessful in some cases, leading to increased mean values with a high standard deviation. As was previously hypothesized[1], oxidation of Met^{15} in minigastrin can occur during handling and incubation, which results in loss of receptor affinity and thus, binding and internalization of the highly affine NMGs.

LogD$_{pH\ 7.4}$

Table S6: summary of logD values of ^{177}Lu-labeled NMGs **1–3** in comparison to PP-F11N determined at pH 7.4.

compound	logD$_{pH\ 7.4}$
PP-F11N	-4.06 ± 0.35
NMG **1**	-3.78 ± 0.23
NMG **2**	-4.04 ± 0.24
NMG **3**	-3.95 ± 0.42

Biodistribution Data

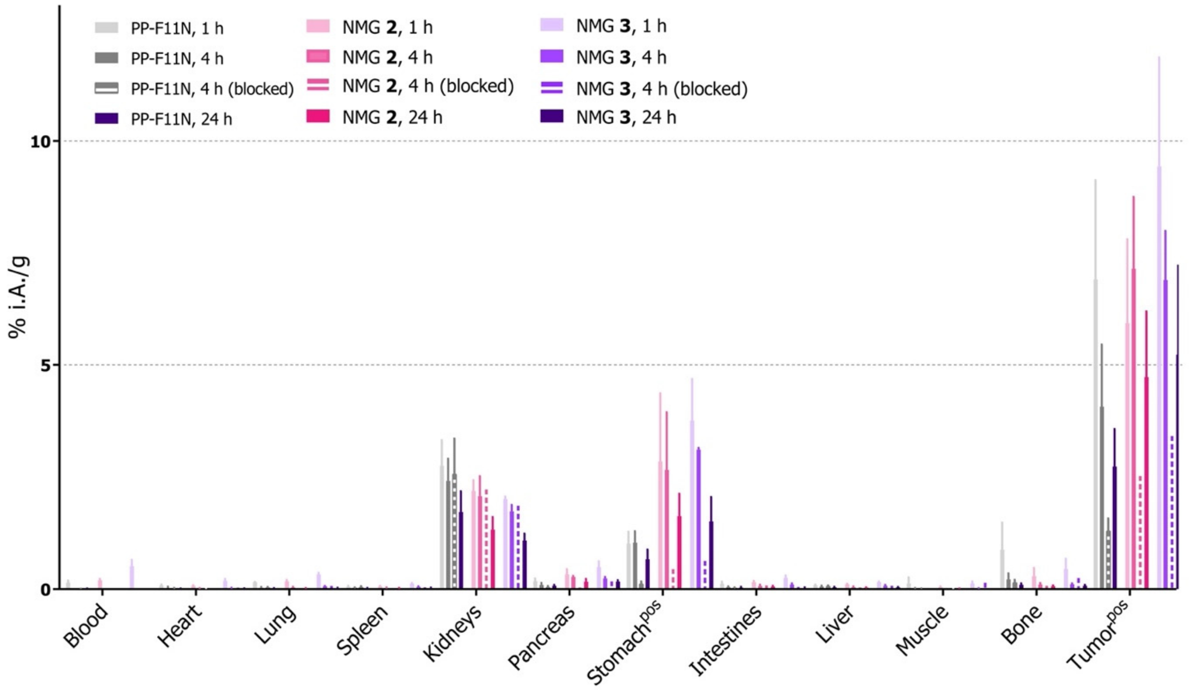

Figure S8: Uptake of all organs for ^{177}Lu-labeled PP-F11N and NMGs **2** and **3** at 1, 4, and 24 h p.i., as well as uptake in presence of a 5000-fold excess of minigastrin at 4 h p.i. (n= 4 animals per group)

Table S7: Biodistribution of [^{177}Lu]Lu-PP-F11N at 1 h, 4 h , 4 h with blocking, and 24 h as mean ± SD. (n = 4 animals per group)

**PP-F11N**	1 h		4 h		4 h (blocked)		24 h	
	% i.A./g	± SD	% i.A./g	± SD	% i.A./g	± SD	% i.A./g	± SD
Blood	0.15	0.04	0.01	<0.01	0.01	0.01	0.01	0.01
Heart	0.08	0.02	0.03	0.03	0.02	0.01	0.01	0.01
Lung	0.16	0.01	0.03	0.01	0.04	0.01	0.02	0.01
Spleen	0.06	0.01	0.03	0.01	0.04	0.03	0.02	0.01
Kidneys	2.75	0.57	2.42	0.49	2.57	0.79	1.72	0.46
Pancreas	0.18	0.06	0.09	0.05	0.04	0.03	0.08	0.02
Stomach^{pos}	1.02	0.26	1.04	0.25	0.12	0.05	0.67	0.22
Intestines	0.12	0.05	0.05	0.01	0.03	0.01	0.03	0.02
Liver	0.09	0.01	0.06	0.02	0.07	0.02	0.04	0.01

Muscle	0.13	0.14	0.02	0.01	0.01	0.01	0.01	<0.01
Bone	0.88	0.60	0.22	0.13	0.15	0.06	0.10	0.03
Tumor^{pos}	6.92	2.21	4.07	1.40	1.30	0.28	2.73	0.84

Table S8: Biodistribution of [^{177}Lu]Lu-NMG **2** at 1 h, 4 h , 4 h with blocking, and 24 h as mean ± SD. (n = 4 animals per group).

**NMG 2**	1 h		4 h		4 h (blocked)		24 h	
	% i.A./g	± SD	% i.A./g	± SD	% i.A./g	± SD	% i.A./g	± SD
Blood	0.20	0.03	0.01	<0.01	0.01	<0.01	<0.01	<0.01
Heart	0.07	0.01	0.02	0.01	0.02	0.01	0.01	<0.01
Lung	0.18	0.03	0.04	0.01	0.04	0.01	0.02	0.01
Spleen	0.06	0.01	0.03	0.01	0.03	<0.01	0.02	0.01
Kidneys	2.19	0.24	2.07	0.45	2.24	0.31	1.33	0.28
Pancreas	0.33	0.11	0.28	0.02	0.06	0.03	0.17	0.06
Stomach^{pos}	2.85	1.52	2.66	1.28	0.46	0.32	1.63	0.50
Intestines	0.15	0.03	0.07	0.03	0.10	0.09	0.06	0.02
Liver	0.11	0.01	0.05	0.01	0.06	0.01	0.03	0.01
Muscle	0.05	0.01	0.01	<0.01	0.02	0.01	0.01	0.01
Bone	0.29	0.18	0.10	0.03	0.09	0.01	0.07	0.02
Tumor^{pos}	5.94	1.87	7.15	1.60	2.54	0.57	4.73	1.47

Table S9: Biodistribution of [^{177}Lu]Lu-NMG **3** at 1 h, 4 h , 4 h with blocking, and 24 h as mean ± SD. (n = 4 animals per group)

**NMG 3**	1 h		4 h		4 h (blocked)		24 h	
	% i.A./g	± SD	% i.A./g	± SD	% i.A./g	± SD	% i.A./g	± SD
Blood	0.51	0.14	0.02	<0.01	0.03	0.03	<0.01	<0.01
Heart	0.19	0.04	0.03	0.01	0.05	0.02	0.01	0.01
Lung	0.33	0.03	0.07	0.01	0.09	0.02	0.03	0.01
Spleen	0.13	0.01	0.05	0.01	0.06	0.01	0.0	0.01
Kidneys	2.01	0.05	1.74	0.14	1.88	0.37	1.09	0.15
Pancreas	0.49	0.13	0.24	0.03	0.19	0.07	0.17	0.029
Stomach^{pos}	3.76	0.93	3.11	0.04	0.65	0.47	1.51	0.54
Intestines	0.25	0.05	0.10	0.02	0.07	0.02	0.02	0.02
Liver	0.16	0.02	0.07	0.02	0.09	0.01	0.04	0.01
Muscle	0.13	0.04	0.02	0.01	0.16	0.24	0.01	<0.01
Bone	0.45	0.22	0.11	0.02	0.26	0.14	0.07	0.02
Tumor^{pos}	9.43	2.43	6.90	1.09	3.43	0.48	5.24	1.99

Table S10: Selected tumor-to-nontumor ratios for ^{177}Lu-labeled PPF11N at 1. 4. and 24 h. (n = 4 animals per group)

Tumor-to	PP-F11N 1 h	PP-F11N 4 h	PP-F11N 24 h
Blood	48.06 ±6.55	406.6 ± 110.1	1038 ± 752
Kidneys	2.53 ± 0.43	1.74 ± 0.55	1.75 ± 0.79
Stomach^{pos}	7.41 ± 3.55	4.09 ± 1.36	4.39 ± 1.47
Muscle	106.6 ± 71.8	355.7 ± 190.3	273.3 ± 65.7

Table S11: Selected tumor-to-nontumor ratios for ^{177}Lu-labeled NMGs **2** and **3** at 1. 4. and 24 h. (n = 4 animals per group)

Tumor-to	NMG **2**<br>1 h	NMG **2**<br>4 h	NMG **2**<br>24 h	NMG **3**<br>1 h	NMG **3**<br>4	NMG **3**<br>24 h
Blood	31.1 ± 10.26	715.4 ± 132.7	4256 ± 1691	19.49 ± 7.58	345.1 ± 34.6	4368 ± 541
Kidney	2.76 ± 0.79	3.50 ± 0.54	3.78 ± 1.48	4.7 ± 1.2	3.99 ± 0.43	3.65 ± 0.99
Stomach^{pos}	2.68 ± 1.57	3.08 ± 1.10	3.13 ± 1.28	2.66 ± 1.08	2.22 ± 0.2	3.06 ± 1.44
Muscle	123.5 ± 42.1	715.4 ± 132.7	440.8 ± 73.7	79.0 ± 15.7	505.2 ± 157.5	436.8 ± 54.1

Table S12: Tumor washout of ^{177}Lu-labeled PP-F11N, NMGs **2**, and **3**.

	**PP-F11N**	NMG **2**	NMG **3**
Tumor 1 h (% i.A./g) ± SD	6.92 ± 2.21	5.94 ± 1.87	9.43 ± 2.43
Tumor 4 h (% i.A./g) ± SD	4.07 ± 1.40	7.15 ± 1.60	6.90 ± 1.09
Relative to 1h	*59%*	*120%*	*73%*
Tumor 24 h (% i.A./g) ± SD	2.73 ± 0.84	4.73 ± 1.47	5.24 ± 1.99
Relative to 1h	*39%*	*80%*	*56%*

Table S13: Two-way analysis of variance (ANOVA) of reduction of uptake of radioactivity in receptor-positive tumor and stomach by co-injection of excess amount of minigastrin. Significance and p-values according to Bonferroni multiple comparisons test performed by GraphPad Prism 8.1.2.

	**PP-F11N**	NMG **2**	NMG **3**
Tumor			
Significant?	Yes	Yes	Yes
P-value	<0.0001	<0.0001	<0.0001
Stomach			
Significant?	No	Yes	Yes
P-value	0.2824	0.0246	0.0004

Additional SPECT/CT

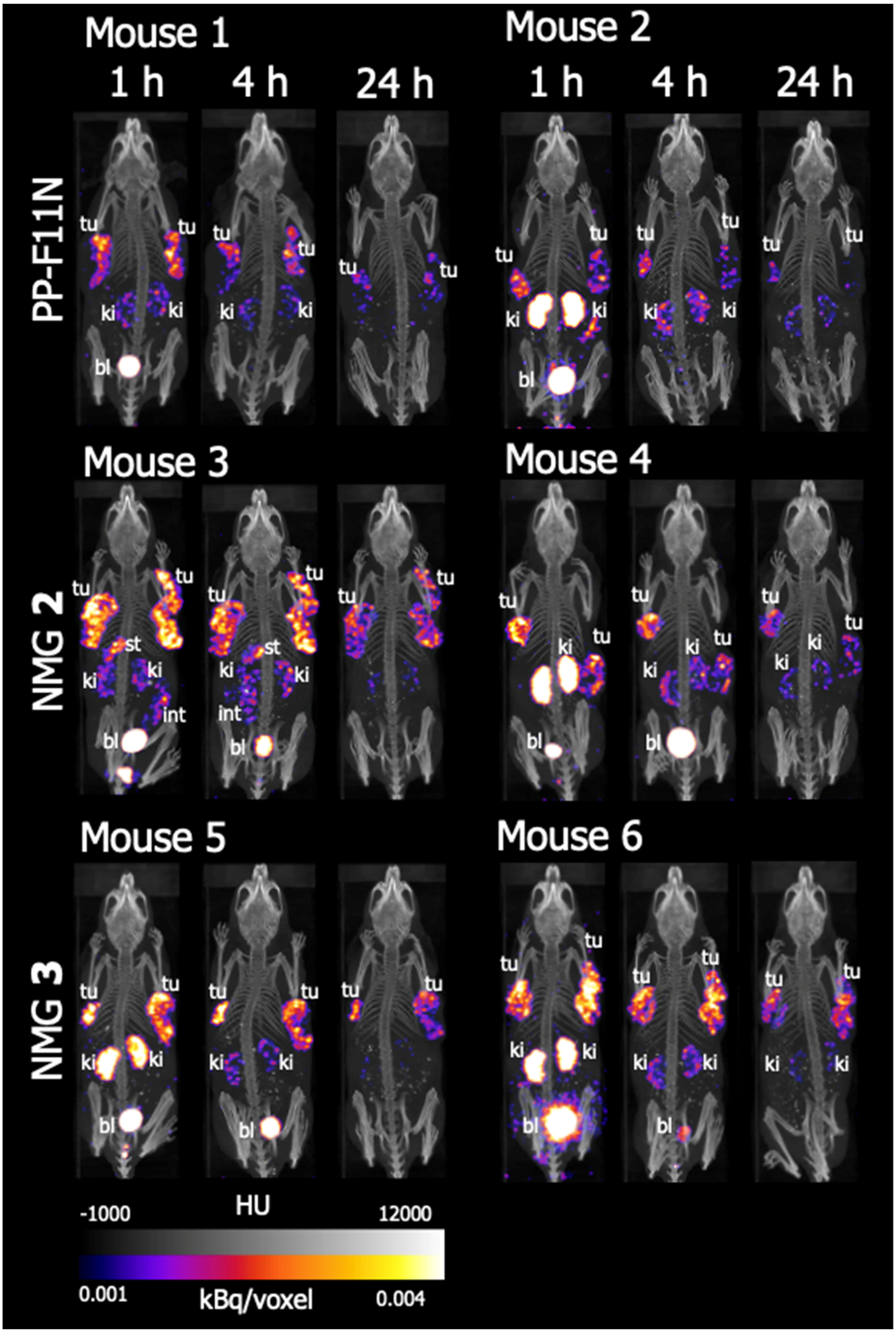

Figure S9: SPECT/CT (MIP) of mice 1-6 with tumor xenografts at 1, 4, and 24 h p.i. of ^{177}Lu-labeled PP-F11N (top row), NMG **2** (middle row), and NMG **3** (bottom row). Color gradient represents intensities from 0.001 (dark purple) to 0.004 (light yellow) kBq/voxel of SPECT, black and white gradient refers to -1000 (black) to 12000 (white) Hounsfield units (HU) of CT. Abbreviations used: tu = tumor xenograft, ki = kidney, bl = urinary bladder, int = intestines. Injections: 200 pmol, 100 μL, 2 μM, 20 MBq, 20 μg/kg. n = 2 animals per compound.

References

1. Grob, N.; Häussinger, D.; Deupi, X.; Schibli, R.; Behe, M.; Mindt, T. L., Triazolo-Peptidomimetics: Novel Radiolabeled Minigastrin Analogs for Improved Tumor Targeting. **2020,** *63*, 4484-4495.

2. Grob, N.; Schmid, S.; Schibli, R.; Behe, M.; Mindt, T. L., Design of Radiolabeled Analogs of Minigastrin by Multiple Amide-to-Triazole Substitutions. **2020,** *63*, 4496.

3. Behe, M.; Schibli, R. Mini-Gastrin Analogue, in Particular for Use in CCK2 Receptor Positive Tumour Diagnosis and/or Treatment. WO2015067473 A1, 2015.
